# SPARKing: Sample-size planning after the results are known

**DOI:** 10.3389/fnhum.2023.912338

**Published:** 2023-02-22

**Authors:** Kyoshiro Sasaki, Yuki Yamada

**Affiliations:** ^1^Faculty of Informatics, Kansai University, Takatsuki, Japan; ^2^Faculty of Arts and Science, Kyushu University, Fukuoka, Japan

**Keywords:** open science, pre-registration, questionable research practices, registered reports, research transparency, sample size

## Introduction

Most psychological research using experimental approaches with null hypothesis significance testing assumes a population, selects a sample from it, and conducts statistical tests on the sampled data. How sample sizes are determined before experiments is crucial for discussing the research's inferential goals through statistical hypothesis testing. Several methods of sample size justification/planning have been proposed (e.g., Kovacs et al., 2022; Lakens, 2022; Maier and Lakens, 2022). Researchers should determine the sample size based on the justifications offered before collecting the data.^1^ Furthermore, they should discuss the results with respect to their sample size justifications. Moreover, based on these justifications, readers evaluate how informative the results are. Sample size justification is one of the factors used to determine what claims can be made based on the study's results and support its reliability.

On the other hand, studies exist that have not justified (or could not justify) the sample size. If honestly reported, lacking a sample size justification may be benign (Lakens, 2022). However, a sample size justification is frequently required according to submission guidelines or reviewers' comments. As a result, manuscripts sometimes receive unfair evaluations simply because they lack sample size justification.

Therefore, cases exist in which retrospective sample size justification is warranted. This strategy is called Sample-size planning after the results are known (SPARKing). SPARKing potentially damages the experimental design's credibility. Thus, it may prevent the appropriate interpretation and reproducibility of the experiment results. This paper reviews cases when SPARKing is questionable and proposes possible solutions.

## When SPARKing is questionable

### Defending results already obtained with a small sample size

Suppose, for example, that a researcher conducted a direct replication experiment to examine a previous study's findings showing a between-participants difference in weight perception between linguistic labels A (“heavy”) and B (“light”) on the surface of an object to be lifted. After they recruited participants from their pool without determining a specific sample size, 20 participants came at a convenient time. They were assigned to each group (*N* = 10 per group). At this time, the researcher analyzed the data, obtained desirable results, and reported them in a paper titled “The effect of linguistic labels on weight perception,” having obtained a significant difference [*t*_(17.6)_ = 2.11, *p* = 0.049, Cohen's *d* = 0.95]. However, during the peer review process, reviewers pointed out the lack of a sample size justification required by the submission guidelines. For this and other reasons, the first journal rejected the manuscript.

The researchers then decided to use SPARKing before submitting to the next journal to increase the possibility of acceptance. In the manuscript, they created a new section called “Sample Size Design.” They declared the following: We chose 10 participants per group as previous studies on weight perception have traditionally used a small sample size (e.g., Marsden et al., 1979; Amazeen and Turvey, 1996). Furthermore, we could not involve a large number of participants in the laboratory due to the COVID-19 lockdown. Furthermore, owing to an abundance of caution, they registered a protocol with Open Science Framework describing this sample size determination method. They included its URL in the same-named section of the manuscript (i.e., Sample Size Design). As a result, the reviewers found no fault with the sample size. Therefore, their paper was accepted for publication in the next journal. Congratulations!

As seen above, nothing essential about the sample size changed. SPARKing has adjusted only superficial credibility. The sample size was justified by other reasons, although it was obtained through the stopping rule based on the researcher's convenience (i.e., the results supported their idea). Here, the researchers used two justification patterns listed by Lakens (2022) resource constraints and heuristics. The previous studies cited were chosen with great precision, although, in fact, the sample size of weight perception studies varies widely. However, many readers may have similar experiences with their work, especially in pandemic conditions.

### Concealing how significant results were obtained by increasing *N*

Suppose that the researchers in Case 1 conducted a new experiment to examine a correlation between the perceived heaviness of a coin (¥500) and mental arithmetic performance. For this study, they decided to perform an a *priori* power analysis. First, they calculated the results with a medium effect size (*r* = 0.3) and 1 – β = 0.80, as they could not predict the effect size. With a one-tailed test, they expected a positive correlation, indicating a required sample size of 64 participants. They collected data on this number of participants. Unfortunately, the results were not significant (*p* = 0.08). They could have discontinued data collection at this point, assuming that a significant trend (or marginally significant results) had been obtained. However, the results appeared too weak to publish.

They suspected that the correlation might become significant if they slightly increased *N*. Therefore, they collected more data. They reasoned among themselves that “in fact, the direction of the predicted relationship was not known a priori,” They modified the test to two-tailed and re-performed a power analysis, which indicated e required sample size of 82 participants. Therefore, they added data until they obtained that number. Regrettably, again their results were not significant (*p* = 0.07). They then reverted the test to one-tailed, set the 1 – β to 0.90, and repeated the power analysis, which indicated that 88 participants were required. They repeated this pattern of modifications, obtaining a similar trend (*p* = 0.07). Thus, they continued to finely tune the effect size, power, and direction of the test for various reasons. Finally, when they finally obtained 614 participants with *r* = 0.1, 1 – β = 0.80, and a one-tailed setting, they found a significant correlation (*p* = 0.04). Fortunately, their survey was online using crowdsourcing, making it relatively simple to increase the sample size. They pre-registered with this sample size design and described only this last configuration in the same-named section of the manuscript, offering a “good” reason. The paper was successfully published. Congratulations!^2^

Here, the researchers used *p*-hacking by increasing *N* (Simmons et al., 2011) while providing justification. When the sample size increases and multiple tests are performed, significant results can almost always be obtained, even with randomly-drawn data (Albers, 2019). The researchers tried out many possible settings in succession within a justifiable range. They stopped adding data at the point where significant results were obtained and reported only the final results and settings. Yet, for many readers, the account provided above sounds quite familiar.

Both cases described above are similar to another questionable research practice (QRP) dedicated to pre-registration called PARKing. PARKing is an abbreviation for pre-registration after the results are known. When researchers engage in PARKing, their behavior severely diminishes the meaning of pre-registration. Their work gains only superficial credibility (Yamada, 2018; Parsons et al., 2022). SPARKing and PARKing work well together because sample size design is always calculated during pre-registration. PARKing is an egregious misuse of the pre-registration system. It is a false representation of a plan executed *post hoc*. Moreover, “adding a description” of the sample size justification to the manuscript after submission is perfectly acceptable, if needed, in cases where it was, in reality, decided in advance. A reasonable justification may exist for adjusting the sample size after starting data collection. In such a case, the researchers might have no problem if they honestly state this. Our concern here involves whether *N* was, in reality, decided in advance and if the reader can detect it. SPARKing is the practice of pretending to have justified a sample size when no such justification exists. Revising the manuscript to include sample size justification is not in and of itself a problem.

## Solutions?

How can we detect SPARKing? First and foremost, authors should not engage in this QRP. As Lakens (2022) noted, disclosing “no justification” (or “resource constraints”) is useful for readers. If authors do not have a specific, legitimate *a priori* design, they should honestly state it up-front.

Furthermore, what are our other options when we cannot expect authors to engage in honest research practices? First, if questionable manuscripts or papers have corresponding preprints, they can be checked for differences in sample size justifications. However, this method is useless when no corresponding preprint exists, if it has been uploaded after masking, or if the researcher recently has added a description of a sample size justification having omitted it initially. Second, regarding the use of SPARKing combined with PARKing (Yamada, 2018; Parsons et al., 2022), differences in timestamps between pre-registrations and all unprocessed data files in a repository might help in efforts to detect these QRPs. However, if unprocessed data files are unavailable or the file's timestamp has been forged or tampered with in some way (e.g., changing the operating system date), this method will also be futile. Thus, detecting SPARKing remains difficult. Studies in which SPARKing has occurred may feature odd sample and effect sizes related to domain knowledge. Thus, readers, including reviewers and editors, should carefully interpret reported values. Even if SPARKing and other QRPs were not executed, false negatives and positives might be found when focusing exclusively on *p*-values.

However, a way to deter SPARKing may exist. Peer-reviewed pre-registration (i.e., the Registered Reports system: e.g., Nosek and Lakens, 2014; Nosek et al., 2018) appears to be a promising solution. Under this system, a manuscript containing only protocols (i.e., protocol manuscript) is peer-reviewed before data collection. Researchers collect data after the protocol manuscript has passed peer review. Notably, researchers will then be required to conduct their experiments and data analyses in line with the accepted protocol manuscript. The sample size justifications are required to be disclosed in the protocol manuscript and cannot be altered after its acceptance, thus preventing SPARKing. As the Registered Reports system is a PARKing countermeasure (Yamada, 2018; Ikeda et al., 2019), it is also a strong prevention method.

## Concluding remarks

This paper aimed to review a new QRP related to the sample size justification, SPARKing. SPARKing potentially damages research credibility. Specifically, SPARKing, in conjunction with hacking pre-registration (i.e., PARKing: Yamada, 2018; Parsons et al., 2022), seems to “unreasonably” boost study reliability. At this time, no effective way to detect SPARKing is available. Of course, encouraging honest reporting of sample size justifications is desirable. Key ways to prevent the QRPs, including SPARKing, are transparency and honesty in reporting. If authors do not have a specific and legitimate prior design related to establishing optimum sample size, they should disclose “no justification” (Lakens, 2022). Moreover, if deviations from the research plan occur, the investigators should report them honestly and describe their reasons. We hope this paper will stimulate discussion within the research community concerning assessing the severity of SPARKing and seeking possible solutions.

## Author contributions

KS: conceptualization, funding acquisition, project administration, resources, writing—original draft, and writing—review and editing. YY: conceptualization, funding acquisition, supervision, resources, writing—original draft, and writing—review and editing. Both authors contributed to the article and approved the submitted version.

## Footnotes

^1^Some exceptions exist. Frequently, researchers determine stopping rules for data collection without sample size justifications. Moreover, when using a Bayesian approach, researchers are allowed to determine whether to collect more data after checking the results.

^2^Please note that adding participants would not necessarily increase the false positive rate, for example, when *p*-values are close to the significance level (Murayama et al., 2014; Tyler, 2022). Even in this case, it is desirable to report the procedures clearly in the manuscript.

## References

[B1] Albers C. (2019). The problem with unadjusted multiple and sequential statistical testing. Nat. Commun. 10, 1921. 10.1038/s41467-019-09941-0 31015469 PMC6478696

[B2] Amazeen E. L. Turvey M. T. (1996). Weight perception and the haptic size-weight illusion are functions of the inertia tensor. J. Exp. Psychol. Human Percep. Perform. 22, 213–232. 10.1037/0096-1523.22.1.213 8742263

[B3] Ikeda A. Xu H. Fuji N. Zhu S. Yamada Y. (2019). Questionable research practices following pre-registration. Japanese Psychol. Rev. 62, 281–295. 10.31234/osf.io/b8pw9

[B4] Kovacs M. van Ravenzwaaij D. Hoekstra R. Aczel B. (2022). SampleSizePlanner: a tool to estimate and justify sample size for two-group studies. Adv. Meth. Pract. Psychol. Sci. 5, 25152459211054059. 10.1177/25152459211054059

[B5] Lakens D. (2022). Sample Size Justification. Collabra: Psychol. 8:33267. 10.1525/collabra.33267

[B6] Maier M. Lakens D. (2022). Justify Your Alpha: A Primer on Two Practical Approaches. Adv. Meth. Pract. Psychol. Sci. 5, 25152459221080396. 10.1177/25152459221080396

[B7] Marsden C. D. Rothwell J. C. Traub M. M. (1979). Effect of thumb anaesthesia on weight perception, muscle activity and the stretch reflex in man. J. Physiol. 294, 303–315. 10.1113/jphysiol.1979.sp012931 512948 PMC1280558

[B8] Murayama K. Pekrun R. Fiedler K. (2014). Research practices that can prevent an inflation of false-positive rates. Pers. Soc. Psychol. Rev. 18, 107–118. 10.1177/1088868313496330 23965303

[B9] Nosek B. A. Ebersole C. R. DeHaven A. C. Mellor D. T. (2018). The preregistration revolution. Proc. Natl. Acad. Sci. 115, 2600–2606. 10.1073/pnas.1708274114 29531091 PMC5856500

[B10] Nosek B. A. Lakens D. (2014). Registered reports: a method to increase the credibility of published results. Soc. Psychol. 45, 137–141. 10.1027/1864-9335/a000192

[B11] Parsons S. Azevedo F. Elsherif M. M. Guay S. Shahimet O. N. Govaart G.. (2022). A community-sourced glossary of open scholarship terms. Nat. Human Behav. 6, 312–318 10.1038/s41562-021-01269-4 35190714

[B12] Simmons J. P. Nelson L. D. Simonsohn U. (2011). False-positive psychology: Undisclosed flexibility in data collection and analysis allows presenting anything as significant. Psychol. Sci. 22, 1359–1366. 10.1177/0956797611417632 22006061

[B13] Tyler C. (2022). On the power and legitimacy of follow-up testing. Perception 51, 225–229. 10.1177/03010066221081204 35285733 PMC8958556

[B14] Yamada Y. (2018). How to crack pre-registration: Toward transparent and open science. Front. Psychol. 9, 1831. 10.3389/fpsyg.2018.01831 30319516 PMC6168681

